# The Effect of Number and Presentation Order of High-Constraint Sentences on Second Language Word Learning

**DOI:** 10.3389/fpsyg.2016.01396

**Published:** 2016-09-15

**Authors:** Tengfei Ma, Ran Chen, Susan Dunlap, Baoguo Chen

**Affiliations:** ^1^Beijing Key Laboratory of Applied Experimental Psychology, School of Psychology, Beijing Normal UniversityBeijing, China; ^2^State Key Lab of Cognitive Neuroscience and Learning, Beijing Normal UniversityBeijing, China; ^3^Department of Education Management, School of Education, The Open University of ChinaBeijing, China; ^4^Children's Learning Institute, University of Texas Health Science Center at HoustonHouston, TX, USA

**Keywords:** second language, word learning, sentence constraint, presentation order, reading

## Abstract

This paper presents the results of an experiment that investigated the effects of number and presentation order of high-constraint sentences on semantic processing of unknown second language (L2) words (pseudowords) through reading. All participants were Chinese native speakers who learned English as a foreign language. In the experiment, sentence constraint and order of different constraint sentences were manipulated in English sentences, as well as L2 proficiency level of participants. We found that the number of high-constraint sentences was supportive for L2 word learning except in the condition in which high-constraint exposure was presented first. Moreover, when the number of high-constraint sentences was the same, learning was significantly better when the first exposure was a high-constraint exposure. And no proficiency level effects were found. Our results provided direct evidence that L2 word learning benefited from high quality language input and first presentations of high quality language input.

## Introduction

Reading is an important way of word learning for adults, especially for adults who are learning a second language (L2) (Pitts et al., 1989; Horst et al., 1998; Zahar et al., 2001; Tekmen and Daloǧlu, 2006; Berwick et al., 2013; Elgort and Warren, 2014). The present study is specifically concerned with L2 word learning through reading.

In studies that explored L2 word learning through reading, some researchers argued that L2 learners need many encounters to learn a word (Horst et al., 1998; Zahar et al., 2001; Waring and Takaki, 2003; Tekmen and Daloǧlu, 2006; Webb, 2008; Pellicer-Sánchez and Schmitt, 2010), thus a topic of importance is how many encounters are needed for L2 learners to learn a new word.

### Number of encounters in L2 word learning

Hulstijn et al. (1996) found little difference between encountering targets words once or three times for Dutchmen who learn French novel words through reading *Menuet*. Horst et al. (1998) suggested at least 8 encounters are needed for their 34 Sudan-English participants who read *The Mayor of Casterbridge* to learn novel words. Webb (2007) suggested more than 10 encounters are needed for English learners from Japan to learn novel words by reading sentences from Oxford Bookworm series. Waring and Takaki (2003) suggested more than 20 encounters are needed for English learners from Japan who read *The Little Prince* to learn the meaning of a novel word.

In summary, the number of encounters needed to learn the meaning of a novel word varied across different studies. One possible reason for this variation could be the proficiency of the L2 learners. Some studies that examined the role of proficiency in L2 word learning found that learners with larger L2 vocabulary size had greater word learning gains through reading and needed fewer encounters (Horst et al., 1998; Zahar et al., 2001; Tekmen and Daloǧlu, 2006). Another reason could be the quality of the language input. Most of these studies above used published novels or stories as reading materials and did not measure or control for complexity of the sentences' syntax and vocabulary. Quality of the language input might be one of the most influential factors of word learning through reading.

### Quality of language input on word learning

Some researchers have explored the quality of language input on word learning (Medina et al., 2011; Cartmill et al., 2013; Trueswell et al., 2013). Cartmill et al. (2013) found that the quality but not the quantity of language input from parents could efficiently predict children's vocabulary 3 years later. And they inferred that the large amount of input might increase the opportunity of encountering high quality situations. Ultimately, it is the quality that truly matters.

Medina et al. (2011) explored how people learn the meaning of words by observing them across different contexts. In their first study, adults watched videos of parents uttering target words within sentences to their infants, and guessed the meaning of the target words which were substituted with a beep. According to these adults' accuracy of guess, they found that about 90% learning instances were uninformative (low quality), and only 7% of instances were informative (high quality), which means in natural situations the quality of most word learning instances are very low. They further investigated the process of word learning with the videos rated in the first study by manipulating the presentation order of high quality contexts in five contexts. Participants were asked to guess the word meaning without any feedback. Response patterns suggested that they made a hypothesis about the meaning of the novel word at the first encounter, and they would maintain the hypothesis at the following learning instances until otherwise disconfirmed by subsequent contradictory information. These results showed that participants'accuracy of guess was significantly higher when high quality context was presented first rather than presented later (trials 2–5), thus indicating the importance of high quality context presented first.

In another study on word learning, they manipulated the number and presentation order of high quality contexts (Trueswell et al., 2013). Participants were presented with two or five visual referents and a spoken sentence containing one novel word per trial. Participants were asked to choose the referent in each trial that they thought matched the novel word, without feedback. Their eye movements were recorded. Researchers found the facilitation effect when the high quality contexts were presented first and the number of high quality contexts was increased. According to the eye movement patterns and accuracy of choice, researchers concluded that the learners made a hypothesis about the word meaning at the first encounter, and then confirmed or rejected upon subsequent encounters. All the results supported their previous findings (Medina et al., 2011), and they named this progression of word learning the “Propose-but-Verify Learning Procedure.”

In Medina and Trueswell's two studies (Medina et al., 2011; Trueswell et al., 2013), the novel word was embedded in an auditory sentence with a video or a picture as a context. They found an effect of number and presentation order of high quality contexts. However, the sentences they used were very simple, such as “Oh look! A zud!.” The sentence itself could not provide much information about the word, but mostly relied on the video or picture to create a high or low quality situation. In word learning through reading, especially for the L2 word learning of adults, the sentences are far more complicated. Because high-constraint sentences provide more information than low-constraint sentences, the quality of sentences could be directly reflected by the sentence constraint.

### Sentence constraint effects on word learning

In the studies of word learning through reading, an important question is how readers make use of sentence context to acquire new words, and whether sentence constraint influences the learning of novel words.

Research from native language word learning through reading found that successful word learning relied on the quality of sentence context; learners could learn more information about novel word from high-constraint sentences than from low-constraint sentences (Borovsky et al., 2010, 2012; Frishkoff et al., 2010).

Frishkoff et al. (2010) trained native English speakers on novel words in high- or low-constraint sentences. Two days after the training, a semantic priming task was applied to test the learners' performance. Event-related potentials (ERPs) were recorded, and the N400 component was analyzed as an indicator of semantic processing. They found larger N400 effects on the words which were seen in high-constraint sentences during the training session, smaller N400 effects on the words which were seen in low-constraint sentences during the training session, and no N400 effects on the words that were never trained.

Borovsky et al. (2010) also examined sentence constraint effects on the understanding and usage of novel words. Twenty-six English native speakers read high-constraint or low-constraint sentences with known or unknown words embedded, and made a plausibility judgment about the word usage in the test sentences. ERPs were recorded during the experiment. Plausibility effects were observed in the N400 component when the novel word was acquired in a high constraint sentence, which demonstrates that native speakers rapidly acquired the novel word usage through high constraint sentences. Borovsky et al. (2012) then investigated the impact of sentence constraint on the integration of novel word meanings into semantic memory. Adult native speakers of English read high-constraint or low-constraint sentences ending with known or unknown words. After reading the sentences, they completed a lexical decision task in which ending words (known or unknown) served as primes for related, unrelated, and synonym target words. ERPs were also recorded during the experiment. They found that N400 amplitudes to target words preceded by unknown word primes varied with prime-target relatedness, but only when the unknown word was embedded in high-constraint sentences previously. The results demonstrated that adult native speakers could rapidly integrate information about word meaning into their mental lexicons by reading high constraint sentences.

These studies so far indicate that native language learners can take advantage of high-constraint sentences to learn new words. Then what about L2 learners? Can they also make use of the sentence constraint to learn new words? There have been some studies exploring the L2 word learning via reading, but many of them were concerned with whether L2 learners could learn new words through natural reading (Pitts et al., 1989; Ellis, 2008), and if so, how many encounters were needed (Horst et al., 1998; Zahar et al., 2001; Waring and Takaki, 2003; Tekmen and Daloǧlu, 2006; Webb, 2008; Pellicer-Sánchez and Schmitt, 2010). Rarely have studies considered whether L2 learners could take use of the differential sentence constraints to learn new words. In the study of Ma et al. (2015), higher and lower-proficiency L2 learners read high or low-constraint sentences to lean novel word meaning. They found L2 learners could take use of high-constraint sentences to lean the meaning of novel words but not the low-constraint. However, the number of sentences was not considered in their study, learner only read one sentence to lean each novel word, and the one sentence was either high or low-constraint. Meanwhile, the presentation order of high-constraint sentences was not investigated in this study.

Therefore, little is known about the effects of number and presentation order of high-constraint sentences on L2 word learning. The present project is devoted to this issue by manipulating the number and presentation order of high-constraint sentences. We strictly controlled variables of the words and sentences, used pseudowords as the learning items and two sentences for each pseudoword as learning instances. The number of high-constraint sentences could be zero (LL) or one (HL and LH) or two (HH), and the first encounter of sentence could be either a high-constraint sentence (HH and HL) or a low-constraint sentence (LH and LL). Because many studies have found that L2 proficiency could make a difference in the second language learning and processing (Horst et al., 1998; Zahar et al., 2001; Ojima et al., 2005; Rossi et al., 2006; Tekmen and Daloǧlu, 2006; Osterhout et al., 2008; Sagarra and Herschensohn, 2010, 2011), we included two groups of L2 learners with different proficiency level (higher and lower).

We predicted that the number of high-constraint sentences would affect the novel word learning performance. More specifically, performance would be better in the HH condition with two high-constraint sentences than in HL or LH condition with only one high-constraint sentences and far better than LL condition with no high-constraint sentences. Also the presentation order of high-constraint sentences would affect the novel word learning performance. Specifically, the performance would be better in HL condition with high-constraint sentences presented first than in LH condition with low-constraint sentences presented first.

## Methods

### Participants

Participants were 49 right-handed college students from Beijing Normal University. All of them had normal or corrected-to-normal vision. The study was approved by the ethics committee of the School of Psychology, Beijing Normal University. All participants signed the written informed consent. They were all native Chinese speakers learning English as a foreign language, who were recruited to our study and split into two groups according to their College English Test (CET) levels. The CET is a proficiency test used to estimate the English level of Chinese college students through listening comprehension, reading comprehension, cloze, error correction, writing, and translation (Zheng and Cheng, 2008). Twenty-five participants who passed CET Band 6 were categorized as higher proficiency English learners; twenty-four participants who failed CET Band 4 were categorized as lower proficiency English learners. Before the experiment, all participants completed self-ratings of their English listening, speaking, reading, and writing abilities on a 5-point scale (1 = very non-proficient, 5 = very proficient), and also completed the Quick Placement Test (Quick Placement Test QPT, 2001), to provide a consistent evaluation of their English level. The QPT is a flexible test of English language proficiency developed by Oxford University Press and Cambridge ESOL to quickly find a student's level of English, including reading and structure, grammar, and vocabulary. Part 1 has 40 items and is taken by all students. Part 2 has 20 items and is administered only to students who did well on Part 1 (Geranpayeh, 2003). All participants of this experiment were asked to complete Part 1 at first, and then Part 2 was administered according to their performance on Part 1. In the end, all participants finished both Part 1 and Part 2. For more details about participants, see Table 1.

**Table 1:** **Background information of participants (***SD***)**.

				**English level self-rating**				
**Participants**	***N***	**Age**	**AoA**	**Listen**	**Speak**	**Read**	**Write**	**QPT**
Higher proficiency	25	22.36 (1.63)	10.40 (1.32)	3.00 (0.82)	3.08 (0.57)	3.68 (0.69)	3.20 (0.71)	47.20 (2.92)
Lower proficiency	24	21.67 (2.30)	11.25 (2.97)	2.17 (0.76)	2.04 (0.69)	3.21 (0.51)	2.75 (0.61)	41.92 (2.24)
*t*-test		1.22	−1.30	3.69^**^	5.75^**^	2.71^**^	2.38^*^	7.09^**^

* p < 0.05;

** p < 0.01; AoA, age of acquisition.

### Experiment design

We adopted a quasi-experimental design, with sentence constraint pair (high-high, high-low, low-high, low-low) as a within-subject factor, and proficiency (higher, lower) as a between-subject factor. The dependent variable was the accuracy of the word production task after sentence reading.

### Materials

#### Real words

Real words were 108 English, high frequency, concrete nouns. Word frequency (mean logFreq = 10.04, *SD* = 0.95) was rated according to HAL norms (Hyperspace Analog to Language Frequency Norms, Lund and Burgess, 1996; Balota et al., 2007). Concreteness (*M* = 578.37, *SD* = 42.54) was rated according to the MRC database (Medical Research Council Psycholinguistic Database, Wilson, 1988). Additionally, familiarity was rated using a 5-point scale (1 = very unfamiliar, 5 = very familiar) by a separate group of 26 college students from the same background as the participants (mean familiarity = 4.83, *SD* = 0.18).

#### Pseudowords

We made 108 pronounceable pseudowords using Wuggy, a multilingual pseudoword generator developed by Keuleers and Brysbaert (2010). This generator uses a specific algorithm to generate pseudowords that can match the subsyllabic structure and transition frequencies with real English words. The length of pseudowords ranged from 5 to 7 letters. These pseudowords were generated from real words (to ensure their pronounceable and rational word formation), yet these real words were not their corresponding target words (108 real words) mentioned above. After all pseudowords were generated, they were randomly matched to the corresponding target words. And no similarities between pseudowords and corresponding target words were found (please see Supplementary Material).

#### Sentences

Four sentences (two high-constraints and two low-constraints) were constructed for each real word, and then we replaced the real word with a pseudoword at random. The length of sentences range from 7 to 17 words, with the key pseudoword always appearing at the end of the sentence (see Table 2). The constraint (high or low) of sentences was rated by another separate group of 43 college students from the same background as the participants. Using a cloze test, these students completed the sentences with the first noun that came to mind. The cloze probability was defined as the percentage of times the same word was provided by these students. The mean cloze probability of high-constraint sentences (85.19%, *SD* = 0.07) was significantly different from low-constraint sentences (12.74%, *SD* = 0.10), [*t*_(215)_ = −89.41, *p* < 0.001, Cohen's d = 6.23].

**Table 2:** **Examples of experimental sentences in different conditions**.

**Constraint pair**	**1st Sentence**	**2nd Sentence**
High-High (HH)	Let's go to the cinema to see a *speath*	The film star Charlie Chaplin was famous for his silent *speath*
High-Low (HL)	Let's go to the cinema to see a *speath*	I am not very interested in that new *speath*
Low-High (LH)	If you are a big fan of him, you will love this *speath*	The film star Charlie Chaplin was famous for his silent *speath*
Low-Low (LL)	If you are a big fan of him, you will love this *speath*	I am not very interested in that new *speath*

To ensure that all sentences were easily understood by our participants, the difficulty degree was rated by the 43 college students who rated the constraint of sentences. Using a 5-point scale (1 = very easy, 5 = very difficult), the overall score was 1.16 (*SD* = 0.07), with no significant difference between high- and low- constraint sentences (high-constraint sentences: *M* = 1.16, *SD* = 0.08; low-constraint sentences: *M* = 1.16, *SD* = 0.06; *t* = −0.74, *p* = 0.46). Sentences were split randomly into four lists to ensure that no items (both real words/pseudowords and sentences) were repeatedly presented in one list, and the real words and their corresponding pseudowords (along with sentences the real words/pseudowords embedded in) were never presented in the same list. Each list included 27 sentences for each of the four conditions (HH, HL, LH, and LL) for a total of 108 sentences. Each participant read only one of the four lists.

### Procedure

Stimuli were presented on a computer using E-prime software version 1.1. We used a whole sentence presentation paradigm. A block of sentences was presented prior to a block of word production tasks that measured learners' behavioral performance. Participants were seated in front of the computer and were provided with instructions and practice trials prior to the experiment. Sentences were presented in a different random order for each participant. Two sentences ending with the same pseudowords were presented as a group, and 6 groups of sentences were presented as a block. Participants read a block of 12 sentences one by one by pressing the space bar on the computer keyboard. When they finished a block of sentences, a “?” prompt would be presented on the screen for 2000 ms to indicate the subsequent word production task. In this task, learners read 6 pseudowords in random order which had just been presented in the 6 groups of sentences and were instructed to write down the meaning of the pseudowords in English on the answer sheet. The accuracy of the word production task was obtained by comparing the answers with the corresponding real words.

After the word production task, all participants were given a checklist of all the materials they had just read to confirm they had no difficulty in reading. For this task, all the pseudowords in the materials were replaced with the corresponding real words. They were asked to mark the words or sentences they felt were hard to understand. As a result, we found no marks on these checklists, which we interpret as an indication that all the materials were reasonably clear to the participants.

## Results

For each of the two proficiency level groups, accuracy of word production task for different conditions is shown in Table 3.

**Table 3:** **Word production accuracy for the higher and lower proficiency level group ***(SD)*****.

	**HH**	**HL**	**LH**	**LL**
Higher proficiency	0.90 (0.30)	0.85 (0.35)	0.83 (0.38)	0.37 (0.61)
Lower proficiency	0.88 (0.32)	0.84 (0.36)	0.79 (0.41)	0.34 (0.47)

HH represents “High-High” condition; HL represents “High-Low” condition; LH represents “Low-High” condition; LL represents “Low-Low” condition.

A mixed-effects logistic model of accuracy was built to analyze the behavior of participants (Baayen et al., 2008; Jaeger, 2008), with constraint pair type and proficiency as fixed factors, subject, and item (combination of sentences and key words) as random factors; and baseline contrasts were performed with HH condition as baseline (Table 4). The model formula was: ACC ~ SentenceType ^*^ Proficiency + (1 | Subject) + (1 | Item). A Tukey *post-hoc* test was applied to reveal the simple main effects of constraint pair. Results are summarized in Table 5. All statistical analyses were carried out using R 3.1.2 (R Core Team, 2014), implemented with package lme4 (Bates et al., 2013), lmerTest (Kuznetsova et al., 2013), and multcomp (Hothorn et al., 2008).

**Table 4:** **Mixed effects logistic model of word production accuracy**.

	**Estimate**	**SE**	***z* value**	***p* (>|z|)**
(Intercept)	2.64129	0.19966	13.229	< 0.001
HH vs. HL	−0.51383	0.17950	−2.863	0.0042
HH vs. LH	−0.79662	0.17507	−4.550	< 0.001
HH vs. LL	−3.35345	0.17084	−19.629	< 0.001
Hp vs. Lp	−0.38586	0.25716	−1.500	0.1335
HL: Lp	0.16633	0.24686	0.674	0.5005
LH: Lp	0.04623	0.23842	0.194	0.8463
LL: Lp	0.33448	0.22810	1.466	0.1425

HH represents “High-High” condition; LH represents “Low-High” condition; LL represents “Low-Low” condition; Hp represents “Higher proficiency”; Lp represents “Lower proficiency.”

**Table 5:** **Tukey ***post-hoc*** test of word production accuracy**.

**Linear Hypotheses**	**Estimate**	**SE**	***z* value**	***p* (>|z|)**
HL–HH	−0.3482	0.1694	−2.0560	0.1954
LH–HH	−0.7506	0.1621	−4.6320	< 0.001
LL–HH	−3.019	0.1582	−19.088	< 0.001
LH–HL	−0.4024	0.1518	−2.6510	0.0468
LL–HL	−2.6708	0.1476	−18.096	< 0.001
LL–LH	−2.2684	0.1377	−16.473	< 0.001

HH represents “High-High” condition; HL represents “High-Low” condition; LH represents “Low-High” condition; LL represents “Low-Low” condition.

As we can see from Tables 4, 5, there was a significant main effect of constraint pair, such that accuracy was higher in the HH condition than in LH condition (*z* = −4.63, *p* < 0.001) and LL condition (*z* = −19.09, *p* < 0.001); higher in the HL condition than in LH condition (*z* = −2.65, *p* = 0.047) and LL condition (*z* = −18.10, *p* < 0.001); higher in the LH condition than in the LL condition (*z* = −16.47, *p* < 0.001). There was no significant difference between the HH and HL condition (*z* = −2.06, *p* = 0.20). No other main effects or interactions were found.

## Discussion

In the present study, we aimed to investigate the effects the effects of number and presentation order of high-constraint sentences on L2 novel word learning. Our results suggested that the number of high-constraint sentences was supportive for L2 word learning, as was shown by the significant differences among HH, LH, and LL conditions. Moreover, when the number of high-constraint sentences was the same, the presentation order of high-constraint sentences played a more important role, as was shown by the significant difference between HL and LH conditions. And when the high-constraint sentences were presented first, the number of high-constraint sentences mattered less, as was shown by the lack of statistical difference between HH and HL conditions.

High-constraint sentences were more supportive for L2 word learning, which is consistent with previous studies on native speakers. Chaffin et al. (2001) used eye-tracking technology to examine how readers establish the meaning of a new word from the sentence context. The sentence context varied in informativeness about the meaning of the new word (informative or neutral). They found that native language learners could make use of the information provided by sentences to infer word meaning. Borovsky and colleagues embedded novel words into sentences with different levels of constraint. They found that in the high-constraint condition, native speakers could acquire the meaning of words through only one exposure (Borovsky et al., 2010, 2012). Our results revealed that one high constraint exposure was sufficient for L2 word learning to occur. Therefore, sentence constraint effects were found both in native language and in second language learners, which suggests that semantic constraint may work similarly in the first and second language. High-constraint sentences could provide more information than low-constraint sentences, which may help readers with inferences when reading sentences and thus facilitated word learning in both first and second language.

More importantly, we found the effects of the number and the presentation order of high-constraint sentences, taking previous findings a step further. Some previous studies about the encounter/exposure times and second language word learning found that with increased encounters, word learning increased (Waring and Takaki, 2003; Tekmen and Daloǧlu, 2006; Webb, 2008; Pellicer-Sánchez and Schmitt, 2010). In our study, each participant read two sentences to learn a novel word. Although the number of encounters for each novel word was the same, there were still differences among different conditions. When the high-constraint sentences were presented more often, participants learned the novel word better. This indicates that the number of encounters was not the primary variable in word learning, it's the number of high-constraint sentences encounters that matters. When the number of encounters increase, so does the possibility of encounters in high quality contexts (e.g., high-constraint sentences in our study), which increased the value of high-constraint sentences on word learning.

Besides the number of high-constraint sentences, the presentation order of high-constraint sentences also affects word learning. Our findings indicated that word meaning abstraction mainly relied on high-constraint sentences, and the order effect indicated the first encounter's importance. As suggested by Medina and colleagues (Medina et al., 2011; Trueswell et al., 2013), the first encounter with a novel word may give learners a hypothesis or guess, and then the following encounter provided either further confirmation or rejection. Results in the present study supported this view to some extent; the first sentences' constraints were crucial, namely, the order of high-constraint sentences affected the novel word learning through reading. Therefore, the number and presentation order of high-constraint sentences are both important facets of the quality of language input on L2 word learning.

No proficiency level effects (main effect or interaction with number of encounters) were found in this study, which is inconsistent with previous studies on proficiency and L2 word learning through reading (Horst et al., 1998; Zahar et al., 2001; Tekmen and Daloǧlu, 2006). In these previous studies, reading materials were published novels or stories without control of reading difficulty; these natural materials may not be proper for lower proficiency learners, and the reading difficulty could be an important factor that impeded lower proficiency learners' subsequent word learning. They may never know what the novel word means, since they could not fully understand many other words in the sentence. In this study, we strictly controlled the words and sentences to ensure all participants' familiarity of materials, and we also required all the participants to confirm they had no difficulty in reading with a checklist of all materials after the experiment. Thus, minimizing and controlling for sentence difficulty, we found that even learners with lower proficiency could effectively process the sentences to learn novel words as readily as the higher proficiency learners.

Some studies on word learning focused more on the role of short-term memory and word knowledge, and were mostly based on situations without context (Gathercole and Masoura, 2003; Hu, 2003; Gray, 2006; Storkel et al., 2006; Maury and Luotoniemi, 2007; Chen and Cowan, 2009). Word learning in reading context is very common in real life, in both native and second language contexts (Nagy et al., 1987; Krashen, 1989; Mestres-Missé et al., 2007, 2008; Berwick et al., 2013; Onnis and Thiessen, 2013). Therefore, more emphasis should be placed on the mechanism of word learning through reading in future research to explore how high-constraint facilitated word learning and what factors may influence this process.

In the present study, each novel word (pseudoword) was presented in only two sentences. Although much research has found that one-time acquisition occurs during word learning (Borovsky et al., 2010, 2012; Shtyrov et al., 2010; Shtyrov, 2011), more evidence suggests we still need practice and repetition to consolidate and refine word learning (Mestres-Missé et al., 2007, 2008; Medina et al., 2011; Munro et al., 2012; Ramscar et al., 2013). In the future, novel words should be presented multiple times to explore the dynamic process of word meaning acquisition.

Words belong to different parts of speech and are processed differently (Crutch and Warrington, 2005; Mestres-Missé et al., 2010). And since only concrete nouns were investigated in the present study, generalization should be cautiously made and further studies on abstract nouns, verbs, or adjectives would show us more about L2 word learning through sentence reading.

In conclusion, the present study took previous findings a step further by focusing on the effects of number and presentation order of high-constraint sentences on second language word learning through reading. Our results suggested that the number of high-constraint sentences was supportive for L2 word learning except when the high-constraint exposure was presented first (there was no significant difference between the HH and HL presentations). When the number of high-constraint sentences was the same, learning was significantly better when the first exposure was a high constraint sentence (HL was better than LH). The present study provides direct evidence that L2 word learning benefits from high quality language input and first presentations of high-constraint contexts.

## Author contributions

TM and BC designed the experiment; TM collected and performed data analysis and wrote the manuscript. RC, BC, and SD edited and revised the manuscript.

### Conflict of interest statement

The authors declare that the research was conducted in the absence of any commercial or financial relationships that could be construed as a potential conflict of interest.
