# Supplementary material for: The Effect of Number and Presentation Order of High-Constraint Sentences on Second Language Word Learning
Source: Front Psychol. 2016 Sep 15;7:1396. doi: 10.3389/fpsyg.2016.01396 (PMC5024562; doi:10.3389/fpsyg.2016.01396)
Supplement: Supplementary file 1 [file Table1.DOCX]

**Appendix**

| **No.** | **Real Word** | **Two Low-constraint Sentences** | **Two High-constraint Sentences** |
| --- | --- | --- | --- |
| **1** | apple | The child reached out a hand towards the ***viboon.*** | The bad queen asked Snow White to eat the poison ***viboon.*** |
|  |  | Her face turns red which looks like a ***vibbon.*** | Isaac Newton discovered gravity through the fall of ***viboon.*** |
| **2** | army | I got this job when I left the ***refite.*** | During the first World War, he joined the ***refite.*** |
|  |  | I spent many unforgettable days with them in the ***refite.*** | Soldiers must obey orders unconditionally in the ***refite.*** |
| **3** | artist | Talent and hard work make her a top ***victap.*** | She studied art and dreamed of becoming a famous ***victap.*** |
|  |  | He couldn't support himself working as a ***victap.*** | The museum exhibition will present works of a new ***victap.*** |
| **4** | audience | He bowed to express his thanks to the ***tonnas***. | The speaker gave a talk which impressed the ***tonnas.*** |
|  |  | As a hostess, her program attracted many ***tonnas***. | Her performance won great applause from the ***tonnas.*** |
| **5** | author | Tom is quite familiar with the well-known ***revasm.*** | For all these years, Lu-Xun has been my favorite ***revasm.*** |
|  |  | I'm very excited to meet my favorite ***revasm***. | We should group together all the books written by the same ***revasm.*** |
| **6** | banana | She was losing weight, only eating milk and ***plare.*** | I slipped on the peel of a ***plare.*** |
|  |  | In this area, they plant many ***plare.*** | A monkey's favorite fruit is the ***plare.*** |
| **7** | bank | There's a nine-floor office building next to the ***sevie.*** | He saves half of his salary in the ***sevie.*** |
|  |  | After graduation, she got a job in the ***sevie.*** | It is safe to keep money in the ***sevie.*** |
| **8** | bear | We were frightened to death by a giant ***sneam.*** | She got a toy as present, it's a Teddy ***sneam.*** |
|  |  | Although I heard a lot about it, I never met a real ***sneam.*** | In Disney cartoon, Winnie the Pooh is a lovely ***sneam.*** |
| **9** | beer | Doctor told him to stay away from ***mubbon.*** | He got drunk on only two glasses of ***mubbon.*** |
|  |  | His father sent him to buy some ***mubbon.*** | You should stay away from alcohol, no wine or ***mubbon.*** |
| **10** | bell | I was so focused that I ignored the ***notid.*** | Turn down the TV; I can't hear the door ***notid.*** |
|  |  | They stopped playing games when they heard the ***notid.*** | The class is over with the sound of ***notid.*** |
| **11** | bird | He is a PhD student who studies on ***ricket.*** | I want to fly, just like a little ***ricket.*** |
|  |  | She opened the window and found a wounded ***ricket.*** | The cat climbed onto the tree to catch the ***ricket.*** |
| **12** | boat | The boy used wood to make a small ***guilor.*** | Without a bridge, they have to cross the river by ***guilor.*** |
|  |  | Driving a car is not the same as driving a ***guilor.*** | We set out to the island by taking a ***guilor.*** |
| **13** | bone | Button can be made of glass, plastic or ***mucket.*** | The human skeleton consists of 206 ***mucket.*** |
|  |  | In Chinese, people love to make soup with ***mucket.*** | Two dogs were fighting over a ***mucket.*** |
| **14** | books | Almost every week, he will buy some new ***jirys.*** | She loves reading; her room is filled with ***jirys.*** |
|  |  | These two kids made a deal about exchanges of ***jirys.*** | He often went to the library to borrow ***jirys.*** |
| **15** | bread | They went from house to house, begging for ***cucket.*** | They walk to the bakery to buy some ***cucket.*** |
|  |  | On the breakfast, I had some orange juice and ***cucket.*** | To make a sandwich you can put meat and vegetables between two slices of ***cucket.*** |
| **16** | bridge | The workers finally complete the construction of the ***redim.*** | Over the river there's a stone ***redim.*** |
|  |  | He dreamed that one day he will be able to bulild a ***redim.*** | The boat is passing under the ***redim.*** |
| **17** | camera | Please show me how to use this new ***jetter.*** | They photographed the fashioned model using a digital ***jetter.*** |
|  |  | We'll go to the zoo this afternoon, and can we borrow your ***jetter?*** | These photos were all taken by his newly bought ***jetter.*** |
| **18** | chair | He put his hat and coat on a ***bindom.*** | In this classroom, the teacher has the biggest desk and ***bindom.*** |
|  |  | They moved into a new house, and wanted to buy some new ***bindom.*** | In his small office, there's a table and a ***bindom.*** |
| **19** | church | The passenger gets off the bus at the ***hustack.*** | She is a religious person who goes to ***hustack.*** |
|  |  | Every Sunday, she went to the ***hustack.*** | Most American weddings are held in a ***hustack.*** |
| **20** | city | A serious traffic accident just happened in the ***peapt.*** | A highway linked the small village to the modern ***peapt.*** |
|  |  | Now we lived far away from the ***peapt.*** | To find a better job, she moved from the country to the ***peapt.*** |
| **21** | clock | He charged me thirty yuan for fixing this ***hetper.*** | I woke up on time by the alarm ***hetper.*** |
|  |  | It just stopped working; I'm considering buy a new ***hetper.*** | My child can already tell the time by the ***hetper.*** |
| **22** | cloud | Mom told him there are different shapes of ***pibbit.*** | The rain stopped and the sun came out from behind the ***pibbit.*** |
|  |  | It could rain or snow according to the ***pibbit.*** | The sky is clear without even one ***pibbit.*** |
| **23** | college | She has received a response from that famous ***goftar.*** | He received four years of formal education at ***goftar.*** |
|  |  | We lost contact since he went to ***goftar.*** | He found a good job after graduating from ***goftar.*** |
| **24** | customer | Don't forget, today we will meet a new ***warliz.*** | We should provide the best service for our each ***warliz.*** |
|  |  | You should do everything you can to satisfy the ***warliz.*** | A good salesman should win the trust of the ***warliz.*** |
| **25** | dance | I'd love to the party, but I can't ***scrool.*** | As she heard the song, she began to ***scrool.*** |
|  |  | She never learned how to ***scrool.*** | The girls there can sing and ***scrool.*** |
| **26** | doctor | At the age of 27, he became a ***mocher.*** | Having been seriously ill, he should see a ***mocher.*** |
|  |  | You must try your best to be a good ***mocher.*** | She had a fever, so she made an appointment with her private ***mocher.*** |
| **27** | dog | The child was afraid to go near the ***wellop.*** | He loves animals, and he kept a cat and a ***wellop.*** |
|  |  | After school, he likes to play ball with his ***wellop.*** | A blind man is sometimes guided by a ***wellop.*** |
| **28** | door | The tools you need were put behind the ***gutlin.*** | I've knocked many times, but nobody answer the ***gutlin.*** |
|  |  | There's a dog sitting beside the ***gutlin.*** | He left the meeting room and closed the ***gutlin.*** |
| **29** | dress | She asked father to buy her a new ***peague.*** | Summer is coming, the girl put on her best ***peague.*** |
|  |  | She painted her nail red to match her ***peague.*** | I must lose weight to fit into that beautiful ***peague.*** |
| **30** | dust | He took off coat to shake down the ***swock.*** | In recent years Beijing has experienced more storms with sand and ***swock.*** |
|  |  | It's hard to see the colors with the cover of ***swock.*** | The room needs cleaning up, it's covered with ***swock.*** |
| **31** | egg | Sorting by size and color, they picked some ***vavier.*** | The naughty boy found a bird's nest and broke one ***vavier.*** |
|  |  | On the way home, she bought some vegetables and ***vavier.*** | The famous painter Da Vinci started his learning by drawing ***vavier.*** |
| **32** | eye | In the fight, he got hurt in the ***drist.*** | The wind blew some sand into his left ***drist.*** |
|  |  | They are both Europeans; see the color of their ***drist.*** | Nothing that happened in this class could escape our teacher's ***drist.*** |
| **33** | factory | Before you start, I'll show you around the ***wobtin.*** | Machines are gradually replacing the workers in the ***wobtin.*** |
|  |  | He had worked for 16 years in this ***wobtin.*** | The government should regulate the pollution problem of this ***wobtin.*** |
| **34** | farm | When I was young, I worked on the ***arram.*** | He raised chickens, sheep and cows on the ***arram.*** |
|  |  | He built a new house on the ***arram.*** | He knows well about planting crops because he lives on a ***arram.*** |
| **35** | fire | They can't figure out the cause of the ***nacoan.*** | The firemen saved many people from the ***nacoan.*** |
|  |  | It's dangerous; you should stay away from the ***nacoan.*** | Do not smoke in the forest; it will cause a ***nacoan.*** |
| **36** | fish | The old man doesn't know how to cook ***musin.*** | The cat entered the kitchen following the smell of ***musin.*** |
|  |  | You are so skinny; you should eat more meat and ***musin.*** | They played in the river, and caught several ***musin.*** |
| **37** | flag | At the top of the building there's a z***amper.*** | There are five stars on our Chinese national ***zamper.*** |
|  |  | Each state in United States has an official ***zamper.*** | The soldiers decided to surrender by waving a white ***zamper.*** |
| **38** | floor | The boy swept the dirt off from the ***hoddle.*** | The book fell from the desk to the ***hoddle.*** |
|  |  | When I woke up, I found myself was on the ***hoddle.*** | There's no seat in the room, so he sat down on the ***hoddle.*** |
| **39** | football | Boys in the class decided to buy a ***poltan.*** | David Beckham was a shining star who played ***poltan.*** |
|  |  | To have fun time with friends, we will play tennis or ***poltan.*** | World Cup is a traditional game of ***poltan.*** |
| **40** | forest | When little, I love to play in the ***geadle.*** | Work with others; one tree doesn't make a ***geadle.*** |
|  |  | It's getting dark, you will get lost in the ***geadle.*** | I want to collect mushrooms; let's go to the ***geadle.*** |
| **41** | friends | New to this country, he hasn't got any ***gurrs.*** | They know each other very well as best ***gurrs.*** |
|  |  | No one can live alone in this world without ***gurrs.*** | He is very shy, and not very good at making new ***gurrs.*** |
| **42** | game | We are split into two groups for this ***unigen.*** | There's little chance of his winning the chess ***unigen.*** |
|  |  | If it rains today, they will cancel the ***unigen.*** | The children don't know how to play the ***unigen.*** |
| **43** | garden | I saw some children running about in the ***oribod.*** | She employed two gardeners to look after her ***oribd.*** |
|  |  | The little girl can spend a whole day playing in the ***oribod.*** | She planted plenty of flowers in her ***oribd.*** |
| **44** | gift | They went to the store to pick some ***pestler.*** | This book was given to me as a birthday ***pestler.*** |
|  |  | I couldn't take this expensive ***pestler.*** | She sent us a picture as a wedding ***pestler.*** |
| **45** | glasses | I want to buy a new pair of ***alpelt.*** | He feels like a blind man when not wearing ***adpelt.*** |
|  |  | Do you remember when I broke your ***alpelt?*** | Many junior students are near-sighted and have to wear ***adpelt.*** |
| **46** | guard | He got work in a hotel as a ***lowen.*** | The thief was caught by a security ***lowen.*** |
|  |  | Every day when he left the company, he'd love to have a few words with the ***lowen.*** | It's not safe for the president to walk without a security ***lowen.*** |
| **47** | gun | They knew that he was always carrying a ***rossage.*** | The hunter shot at a deer with his ***rossage.*** |
|  |  | The new soldier doesn't know how to use a ***rossage.*** | The robber ran into a bank and fired his ***rossage.*** |
| **48** | hat | She happened to meet a girl in red ***coddin.*** | To protect your head from cold wind, wear a ***coddin.*** |
|  |  | In winter, he loves to wear a brown ***coddin.*** | A sudden wind blew off her red ***coddin.*** |
| **49** | hospital | I used to work as a cleaner in ***romber.*** | The patient should be sent to ***romber.*** |
|  |  | We are so lucky, not far from here, there is a ***romber.*** | He had a medical check-up in the ***romber.*** |
| **50** | ice | He opened the fridge and get out some ***naswin.*** | Low temperature turns water into ***naswin.*** |
|  |  | She loves to have coffee or tea with ***naswin.*** | When the river freezes, we can walk on the ***naswin.*** |
| **51** | island | Many people dream about living on a beautiful ***pramon.*** | The new bridge links the mainland with the ***pramon.*** |
|  |  | There's a fairy-tale castle on this ***pramon.*** | Spring is with us all the year round on Hainan ***pramon.*** |
| **52** | key | He was looking around trying to find the ***buite.*** | I couldn't open the lock; you've given me the wrong ***buite.*** |
|  |  | She opened the box and found a ***buite.*** | I'm locked out; do you have a spare ***buite.*** |
| **53** | kid | Nothing is afraid of, you are not a ***desuce.*** | I started playing piano when I was a ***desuce.*** |
|  |  | People all said that he was a smart ***desuce***. | I've known you since you were a ***desuce.*** |
| **54** | kiss | His daughter came into his arms for a ***brinny.*** | In the fairytale, Sleeping Beauty woke up with the prince's ***brinny.*** |
|  |  | I felt very excited and I gave her a ***brinny.*** | Every morning, they gave each other a hug and a ***brinny.*** |
| **55** | knife | He tried to frighten me by showing the ***isefy.*** | When cooking, she cut her finger with ***isefy.*** |
|  |  | She murdered her husband with ***isefy.*** | We eat with chopsticks while westerners use a fork and ***isefy.*** |
| **56** | lawyer | He clearly answered questions put forward by the ***rasium.*** | She is studying law, hoping to become a ***rasium.*** |
|  |  | I think you'd better ask advices from a ***rasium.*** | In court, he answered all the questions put forward by the ***rasium.*** |
| **57** | leader | She believes that he will be the future ***ptoter.*** | All members chose him to be their team ***ptoter.*** |
|  |  | He doesn't feel himself qualified to be a ***ptoter.*** | Most members of the party are supporters of the new elected ***ptoter.*** |
| **58** | leg | A month later, the doctor carefully rechecked his ***candot.*** | She preferred to eat the chicken's wing rather than ***candot.*** |
|  |  | I was in pain, and I couldn't move my ***candot.*** | He fell from bike and broke his left ***candot.*** |
| **59** | letter | She was angry that he didn't answer her ***pectute.*** | The boy fell in love with a girl, and he wrote her a love ***pectute.*** |
|  |  | He was so busy that he forgot to send this ***pectute.*** | Attach a stamp before you send the ***pectute.*** |
| **60** | library | I came across an old friend in the ***creath.*** | There's a large collection of books in our school's ***creath.*** |
|  |  | I need to go to the local ***creath.*** | It's the due date; I must return these books to the ***creath.*** |
| **61** | light | You can move the desk to get better ***phosh.*** | It is getting dark, please turn on the ***phosh.*** |
|  |  | In the middle of the ceiling, there's a ***phosh.*** | It is illegal to drive ahead ignoring the red ***phosh.*** |
| **62** | map | Turn this page over and you'll see a ***dusin.*** | Beijing is marked by a red star on the Chinese ***dusin.*** |
|  |  | All we have to do is to follow the ***dusin.*** | I couldn't tell direction without refer to Google ***dusin.*** |
| **63** | medicine | Don't let children get in touch with this ***bribod.*** | She's got a cold, so the doctor prescribed some ***bribod.*** |
|  |  | The girl made a face at the taste of the ***bribod.*** | To cure your disease, you must take the bitter ***bribod.*** |
| **64** | milk | He satisfied his hunger with a sandwich and ***clore.*** | The natural food for young babies is mother's ***clore.*** |
|  |  | Many people love to eat food with coffee, tea or ***clore.*** | To get plenty of calcium and protein, you need to drink ***clore.*** |
| **65** | mirror | Many girls spent hours in front of the ***smider.*** | She was looking at herself in the ***smider.*** |
|  |  | On the wall of their bedroom, there's a ***smider.*** | Who is the fairest in the world? The queen asked the magic ***smider.*** |
| **66** | money | I hope you stop worrying too much about ***zeark.*** | He is rich, but he still wants more ***zeark.*** |
|  |  | Never be a friend with him, since he never returned other's ***zeark.*** | She married the rich man only for his ***zeark.*** |
| **67** | moon | She loves to tell the story about the ***empock.*** | At night you can see stars surround the ***empock.*** |
|  |  | When he was a kid, he often dreamed about getting on the ***empock.*** | We never imagined that men could land on the ***empock.*** |
| **68** | mountain | The river is divided into two by a ***lectode.*** | When the earthquake occurred, snow and rocks fell down the ***lectode.*** |
|  |  | Behind their village, there's a beautiful ***lectode.*** | He fell behind when we were climbing the ***lectode.*** |
| **69** | movie | I am not very interested in that new ***speath.*** | Let's go to the cinema to see a ***speath.*** |
|  |  | If you are a big fan of him, you will love this ***speath.*** | She won the Oscar Award for her performance in a new ***speath.*** |
| **70** | newspaper | She sat down on chair to read a ***swood.*** | News of their marriage was published in today's ***swood.*** |
|  |  | Hey, can you pass me that ***swood?*** | He read about the striking news in the ***swood.*** |
| **71** | nose | Yesterday she accidentally fell down and broke her ***unane.*** | The boy smelled something different with his sensitive ***unane.*** |
|  |  | She had a plastic surgery to change the shape of her ***unane.*** | We could not breathe or smell without the ***unane.*** |
| **72** | novel | She had spent a peaceful afternoon with a ***purder.*** | The novelist told interesting stories in his new ***purder.*** |
|  |  | He gets many criticisms for his new ***purder.*** | This movie was adapted from a famous ***purder.*** |
| **73** | ocean | One day, he went for a swim in the ***bliat.*** | As we know, water is saltwater in the ***bliat.*** |
|  |  | They went on a ship to across the ***bliat.*** | The plane crashed and fell into the ***bliat.*** |
| **74** | oil | There must be something pushing up the price of ***ratid.*** | If the machine can't work smoothly, try adding some ***ratid.*** |
|  |  | When cooking, you should put less salt and ***ratid.*** | Traditional sources of energy include coal, gas and ***ratid.*** |
| **75** | paper | Wood can be used as material for making ***quord.*** | Please write this down on a piece of ***quord.*** |
|  |  | It can't be printed now, since we are out of ***quord.*** | You should prepare yourself with pen, ink and ***quord.*** |
| **76** | passenger | Please pass me the name list of the ***fover.*** | This bus has an empty seat; it can carry one more ***fover.*** |
|  |  | The thief stole a phone and a purse from a ***fover.*** | Only two 30kg luggage were allowed for each ***fover.*** |
| **77** | patient | The news must not be told to the ***hildet.*** | The doctor did his best to cure the ***hildet.*** |
|  |  | He noticed a gradual improvement in this ***hildet.*** | Four nurses were taking care of the seriously ill ***hildet.*** |
| **78** | phone | She had spent all night waiting by the ***cipter.*** | I want to talk with you face to face, not just on the ***cipter.*** |
|  |  | Nowadays, you cannot imagine going out without taking your ***cipter.*** | I need to make a call; may I use your ***cipter.*** |
| **79** | plane | Hurry or you will be late for your ***proft.*** | Passengers must check in at the airport before getting on the ***proft.*** |
|  |  | We waited two hours before we got on the ***proft.*** | We will fly to London by taking the next ***proft.*** |
| **80** | prison | In a remote village, there is a large ***callian.*** | Because of murder, he spent his whole life in ***callian.*** |
|  |  | He spent most time studying during his ten years in ***callian.*** | The thief was caught and sent to ***callian.*** |
| **81** | purse | I forget which place I have put the ***thire.*** | Money and credit cards are gone with my lost ***thire.*** |
|  |  | I think there's someone stole my ***thire.*** | A coin falls out of my ***thire.*** |
| **82** | rat | She felt sick when she saw a dead ***punger.*** | The cat waits beside the hole to catch a ***punger.*** |
|  |  | This store was overrun with ***punger.*** | Tom and Jerry is a famous cartoon in which Jerry is a smart ***punger.*** |
| **83** | restaurant | Tomorrow evening, I have a date at that ***snamp.*** | I don't want to cook, let's go to a ***snamp.*** |
|  |  | This afternoon, I will meet him at the ***snamp.*** | The food is delicious in that ***snamp.*** |
| **84** | rice | This kind of wine is made out of ***candan.*** | People from north China prefer noodles, while southerners prefer ***candan.*** |
|  |  | I'd love to have some fish and ***candan.*** | Even the cleverest housewife can't cook a meal without ***candan.*** |
| **85** | ring | You can never make a proposal without an ***owfan.*** | At the wedding, the bride and groom exchange their ***owfan.*** |
|  |  | When cooking, she always took off the ***owfan.*** | He proposed to his girlfriend with a diamond ***owfan.*** |
| **86** | river | They bought a big house just beside the ***lurple.*** | She killed herself by jumping into a ***lurple.*** |
|  |  | After meal, my parents went for a walk along the ***lurple.*** | Two children drowned after falling into the ***lurple.*** |
| **87** | servant | You must have been an honest and faithful ***vattey.*** | Bring us the menu, she said to the ***vattey.*** |
|  |  | At spare time, many college students worked as ***vattey.*** | To clean his house every day, he hired a ***vattey.*** |
| **88** | shape | These bags differ in size, but not in ***banble.*** | Blind person use their fingers to feel the object's ***banble.*** |
|  |  | This new robot is capable of recognizing different ***banble.*** | Liquid flows freely without a fixed ***banble.*** |
| **89** | sheep | They sent a girl to look after these ***loler.*** | He is a wolf in the clothing of a ***loler.*** |
|  |  | During the night, the thief stole the farmer's ***loler.*** | When you can't sleep, you may try counting ***loler.*** |
| **90** | sister | The color of her hair differs from her ***gusser.*** | I'm the only child, without any brother or ***gusser.*** |
|  |  | He came forward and embraced his ***gusser.*** | She looks almost the same as her twin ***gusser.*** |
| **91** | skin | Honestly, you should pay more attention to your ***tuser.*** | Long time exposure to sun will change the color of your ***tuser.*** |
|  |  | The cold wind will quickly dry out your ***tuser.*** | Snow-White has red lips, black hair and white ***tuser.*** |
| **92** | sky | You may feel free when looking into the ***edlay.*** | The stars are twinkling in the clear night ***edlay.*** |
|  |  | She likes the color of the clear ***edlay.*** | Look, there is a rainbow in the ***edlay.*** |
| **93** | smile | He welcomed me back with a handshake and ***barpit.*** | He is optimistic, always accepting troubles with a ***barpit.*** |
|  |  | She was a pleasant lady with a kind ***barpit.*** | When we feel happy and satisfied, we always ***barpit.*** |
| **94** | snow | In this region, it is unusual to see ***gulct.*** | I like winter best because of the white ***gulct.*** |
|  |  | It was the first time I saw ***gulct.*** | Her skin is as white as ***gulct.*** |
| **95** | socks | It's so smelly, can’t you just wash your ***athits!*** | On Christmas Eve, Saint Claus will put gifts in ***athits.*** |
|  |  | His mom bought him a new pair of ***athits.*** | It's smelly; can't you just wash your feet and ***athits.*** |
| **96** | song | It takes a long time to record a ***lumic.*** | They stand side by side, singing an old ***lumic.*** |
|  |  | He is blind, but he can play many beautiful ***lumic.*** | She has a beautiful voice, and it's perfect for this ***lumic.*** |
| **97** | stone | Look at that house, it is built of ***rallen.*** | He has broken the window by throwing a ***rallen.*** |
|  |  | He couldn't tell whether the floor is wood or ***rallen.*** | The bread is too dry; it's as hard as a ***rallen.*** |
| **98** | storm | The lab meeting was canceled because of the ***bopple.*** | The fishing boat was caught in a heavy ***bopple.*** |
|  |  | The weather forecast predicts that there will be a ***bopple.*** | Light travels faster than sound, so we see lightning first in a ***bopple.*** |
| **99** | sugar | Go down to the grocery and get some ***punpet.*** | He tasted the coffee, then added milk and ***punpet.*** |
|  |  | Many kids love to eat food with ***punpet.*** | It's bad for your teeth to eat too much ***punpet.*** |
| **100** | teacher | He drew a funny picture of the old ***anspar.*** | All students are equal in the eyes of my ***anspar.*** |
|  |  | The naughty boy kept silent and didn't answer his ***anspar.*** | To learn about his performance at school, his parents talked with his ***anspar.*** |
| **101** | teeth | She likes people who with nice and clean ***wartet.*** | After each meal, you should brush your ***wartet.*** |
|  |  | They turned yellow, since he doesn't often clean his ***wartet.*** | The little girl smiled, showing her perfect white ***wartet.*** |
| **102** | tickets | With the help of friend, I got a ***nusks.*** | We decided to travel this weekend, and I've booked 3 flight ***nusks.*** |
|  |  | There're still many people waiting to buy ***nusks.*** | Before getting on the train, passengers need to show their ***nusks.*** |
| **103** | toy | He is curious about how to make a ***mourt.*** | Girls all like Barbie doll, it may be the most famous ***mourt.*** |
|  |  | Every child grows up with ***mourt.*** | It looks like a real dog, but it is just a ***mourt.*** |
| **104** | umbrella | I made a mistake by taking someone else's ***ceague.*** | It's going to rain, remember to bring your ***ceague.*** |
|  |  | I need to buy a new one since I lost my ***ceague.*** | Don't get wet, come under my ***ceague.*** |
| **105** | water | Please hang on, I will go for some ***swink.*** | The doctor just said keep warm and drink more ***swink.*** |
|  |  | To make it clean, I need more ***swink.*** | The plants died from lacking of ***swink.*** |
| **106** | window | There is a young man sitting beside the ***bodmiz.*** | Open the curtain to let light through the ***bodmiz.*** |
|  |  | He noticed his sister standing silently over the ***bodmiz.*** | It's cold outside; please close the door and the ***bodmiz.*** |
| **107** | world | Come on, it isn't the end of the ***quing.*** | When I become rich I'll travel around the ***quing***. |
|  |  | His advanced idea really changed the ***quing.*** | Bill Gates is one of the richest people in the ***quing.*** |
| **108** | zoo | He is waiting for a bus to the ***jalleb.*** | These animals spent their life in the locked iron cage in the ***jalleb***. |
|  |  | Her father promised that this weekend they will go to the ***jalleb.*** | The keeper said a monkey escaped from the ***jalleb.*** |
